# Association between obesity and likelihood of remission or low disease activity status in psoriatic arthritis applying index-based and patient-based definitions of remission: a cross-sectional study

**DOI:** 10.1136/rmdopen-2023-003157

**Published:** 2023-09-14

**Authors:** Ying Ying Leung, Lihi Eder, Ana-Maria Orbai, Laura C Coates, Maarten de Wit, Josef S Smolen, Uta Kiltz, Penélope Palominos, Juan D Canete, Rossana Scrivo, Andra Balanescu, Emanuelle Dernis, Sandra Meisalu, Martin Soubrier, Umut Kalyoncu, Laure Gossec

**Affiliations:** 1 Department of Rheumatology & Immunology, Singapore General Hospital, Singapore; 2 ACP Medicine, Duke-NUS Medical School, Singapore; 3 Department of Medicine, Women's College Research Institute, University of Toronto, Toronto, Ontario, Canada; 4 Medicine Rheumatology, Johns Hopkins University, Baltimore, Maryland, USA; 5 Nuffield Department of Orthopaedics, Rheumatology and Musculoskeletal Sciences, University of Oxford, Oxford, UK; 6 Patient Research Partner, EULAR, Zaltbommel, The Netherlands; 7 Rheumatology, Medical University of Vienna, Vienna, Austria; 8 Rheumazentrum Ruhrgebiet, Herne and Ruhr-Universität Bochum, Bochum, Germany; 9 Division of Rheumatology, Hospital de Clinicas de Porto Alegre, Porto Alegre, Brazil; 10 Rheumatology Department, Hospital Clinic and Institut D'Investigacions Biomediques August Pi Sunyer, Barcelona, Spain; 11 Rheumatology, Sapienza Università di Roma, Rome, Italy; 12 Department of Internal Medicine and Rheumatology, ‘Sf. Maria’ Hospital, Carol Davila University of Medicine and Pharmacy, Bucharest, Romania; 13 Rheumatology, Centre Hospitalier Le Mans, Le Mans, France; 14 Department of Rheumatology, East Tallinn Central Hospital, Tallinn, Estonia; 15 Rheumatology, University Hospital Centre Gabriel Montpied, Clermont-Ferrand, France; 16 Department of Internal Medicine, Division of Rheumatology, Hacettepe University Faculty of Medicine, Ankara, Turkey; 17 INSERM, Institut Pierre Louis d'Epidémiologie et de Santé Publique, Sorbonne Universite, Paris, France; 18 APHP, Rheumatology Department, Hopital Universitaire Pitie Salpetriere, Paris, France

**Keywords:** Arthritis, Psoriatic, Outcome Assessment, Health Care, Patient Reported Outcome Measures

## Abstract

**Objectives:**

We aimed to evaluate whether obese patients with psoriatic arthritis (PsA) were less likely to be in remission/low disease activity (LDA).

**Methods:**

We used data from the ReFlaP, an international multi-centre cohort study (NCT03119805), which recruited consecutive adults with definite PsA (disease duration ≥ 2 years) from 14 countries. Demographics, clinical data, comorbidities, and patient-reported outcomes were collected. Remission/LDA was defined as Very Low Disease Activity (VLDA)/minimal disease activity (MDA), Disease Activity in PSoriatic Arthritis (DAPSA) ≤4/≤14, or by patients’ opinion. Obesity was defined as physician-reported and/or body mass index ≥30 kg/m^2^. We evaluated the association between obesity and the presence of remission/LDA, with adjustment in multivariable regression models.

**Results:**

Among 431 patients (49.3% women), 136 (31.6%) were obese. Obese versus non-obese patients were older, more frequently women, had higher tender joint and enthesitis counts and worse pain, physical function and health-related quality of life. Obese patients were less likely to be in VLDA; DAPSA remission and MDA, with adjusted ORs of 0.31 (95% CI 0.13 to 0.77); 0.39 (95% CI 0.19 to 0.80) and 0.61 (95% CI 0.38 to 0.99), respectively. Rates of DAPSA-LDA and patient-reported remission/LDA were similar for obese and non-obese patients.

**Conclusion:**

PsA patients with comorbid obesity were 2.5–3 folds less likely to be in remission/LDA by composite scores compared with non-obese patients; however, remission/LDA rates were similar based on the patients’ opinion. PsA patients with comorbid obesity may have different disease profiles and require individualised management.

**WHAT IS ALREADY KNOWN ON THIS TOPIC**

- A few Caucasian studies have indicated that patients with obesity may be less likely to achieve remission.

**WHAT THIS STUDY ADDS**

- Congruent with the existing literature, we have shown from an international multicentre study that obesity is associated with higher pain scores and worse physical function and health-related quality of life. Patients with comorbid obesity also had lower probability of the presence of remission/low disease activity (LDA) by composite scores.
- The differences in rates of remission/LDA between obese and non-obese patients according to patients’ opinion were lower.

**HOW THIS STUDY MIGHT AFFECT RESEARCH, PRACTICE OR POLICY**

- This highlights the importance of recognising and managing obesity as an important comorbidity to improve the care of patients with psoriatic arthritis.

## Introduction

Psoriatic arthritis (PsA) is a systemic immune-related rheumatic disease with many clinical manifestations.^1^ In addition to musculoskeletal and skin disease, the key domains relevant to both physicians and patients include physical function, fatigue, patient global assessment (PGA) and health-related quality of life (HR-QoL).^2 3^ PsA is also associated with an increased prevalence of metabolic and cardiovascular comorbidities, such as obesity, hypertension, hyperlipidaemia, type II diabetes and coronary heart disease. According to the treat-to-target management strategy (T2T), achieving remission (or alternatively, low disease activity, LDA) is an important target.^4 5^ Achieving a state of remission or LDA is associated with improved patient-reported outcomes (PROs) and physical function and less radiographic progression.^6^ It may also be associated with lower atherosclerosis burden in the long term.^7^

Some indices have been developed to assess or define states of remission or LDA, including the binary states of Very Low Disease Activity (VLDA) and minimal disease activity (MDA) and the continuous score Disease Activity in PSoriatic Arthritis (DAPSA) with cut-offs for remission and LDA.^8 9^ To date, there is no consensus on which remission target is best to guide treatment in clinical practice.^4^ In addition, these indices may not necessarily reflect the patients’ opinion on their status.^10 11^ We previously reported that the agreement between remission/LDA by composite indices and patients’ opinion was only moderate to good.^10^

Obesity is common among patients with PsA and is an important comorbidity.^12^ Obesity is more prevalent among patients with PsA than in patients with rheumatoid arthritis or the general population. Obesity is a risk factor for the development of psoriasis in the general population,^13 14^ it is also associated with an increased risk of PsA among those with psoriasis.^15^ Obesity may be a consequence of a sedentary lifestyle due to musculoskeletal inflammation and pain or psychological distress from skin disease. Adipokines secreted by the adipose tissue may have pro-inflammatory functions and worsen both skin and joint disease in PsA.

A few studies have indicated that patients with obesity may be less likely to achieve remission.^16 17^ Furthermore, weight reduction may improve the achievement of remission for those initiating biological therapies.^18^ However, little is known on the effect of obesity on reaching remission/LDA when defined by indices and patients’ opinion. In this study, we aimed to explore the association between obesity and remission/LDA when defined according to validated indices and patients’ opinion, using an international clinic cohort.

## Methods

We used the baseline data of an international multicentre study performed in 2018, Remission/Flare in PsA (ReFlaP; NCT03119805).^10^ ReFlaP recruited adult patients with physician diagnosed PsA from 21 centres in 14 countries (Austria, Brazil, Canada, Estonia, France, Germany, Italy, Romania, Russia, Singapore, Spain, Turkey, United Kingdom and USA). We recruited patients with at least 2 years’ disease duration, as they should have a more comprehensive experience of disease flare or remission.

### Data collection

We collected demographic data, including age, sex, years of schooling, current use of conventional synthetic disease-modifying antirheumatic drugs (csDMARDs) and/or biologic (b-)DMARDs. Clinical data collected included 66/68 swollen/tender joint counts, enthesitis (Leeds Enthesitis Index) and C reactive protein (CRP). Comorbidities were collected using the Functional Comorbidity Index (FCI), which comprises 17 additional conditions on top of arthritis.^19^ Recent data have demonstrated preliminary data on construct validity^20^ and known group validity^21^ of FCI in PsA.

PROs included pain (in numeric rating scale, NRS 0–10, none to worst), PGA of disease activity (NRS 0–10, none to worst), Health Assessment Questionnaire-disability index (HAQ-DI) and Psoriatic Arthritis Impact of Disease-12 (PsAID-12).

### Defining remission

Three remission criteria were used in this study.

1. Remission by Disease Activity index for PSoriatic Arthritis (DAPSA-REM). DAPSA is a measure of peripheral arthritis activity with summation of tender joint count (0–68), swollen joint count (0–66), pain, PGA and CRP.^22^ A cut-off value of ≤4 was considered as DAPSA-REM; >4 and ≤14 as LDA; >14 and ≤28 as moderate disease activity and >28 as high disease activity.^23^
2. VLDA, defined as the presence of these seven items: tender Joint count ≤1; swollen joint count ≤1; enthesitis count ≤1; PGA ≤2/10; pain ≤1.5/10; skin psoriasis (PASI ≤1 or BSA ≤3%) and HAQ-DI ≤0.5.^24^
3. Remission by patients’ opinion. We asked patients to answer yes/no to this question: at this time, is your PsA in remission, if this means: you feel your disease is as good as gone? This phrasing of this remission criteria was developed with input from four patient research partners with PsA and based on previous work in remission criteria in rheumatoid arthritis.^25^ We previously reported moderate agreement of remission by patients’ opinion and PGA ≤1 (Kappa 0.43).^10^

LDA data were collected using (1) MDA defined as having at least 5/7 items in VLDA criteria (including VLDA); (2) DAPSA-LDA (inclusive of DAPSA-REM), defined as DAPSA ≤14; and finally, (3) LDA by patients’ opinion, by patients answering ‘yes’ to this question: ‘at this time, are you in LDA, if this means: your disease is in low activity but it is not as good as gone?’

### Obesity

Obesity information was collected by two methods. First, body weight and height were measured at recruitment to calculate the body mass index (BMI) in kg/m^2^. Second, comorbidities were collected by the attending physician as yes/no answers in the FCI obesity item.^19^ Obesity in the current study was defined as a ‘yes’ answer to FCI obesity item and/or measured BMI ≥30 kg/m^2^.

### Statistical analysis

Patients with available data for remission (by indices and patients’ opinion) were included in this analysis. Demographic and clinical variables were described as mean (SD) or frequencies for continuous or categorical variables as appropriate; comparisons were made between patients with obesity versus non-obese. No imputation of missing data was performed; data were analysed on complete cases.

We compared the rates of obesity according to each remission/LDA definition. We constructed generalised linear models (binary logistic type), for the outcome variables, that is, the three remission/LDA definitions. In addition to obesity, variables that were not components of remission criteria were considered to be included as adjustment variables, that is, age, sex, years of schooling, duration of PsA, use of current cs/b-DMARDs and other comorbidities. These variables were chosen as they have previously been reported to be associated with remission in PsA.^16 17 26^Comorbidities other than obesity were considered using the FCI without the obesity category to avoid circularity. Although disease activity variables were the major contributor of remission/LDA as we previously reported,^26^ we did not consider them in analysis models as they are the components of remission/LDA definitions. Variables associated with remission/LDA outcomes with p value ≤0.10 in the univariable models were included in the multivariable logistic regression models. As female sex is known to be associated with remission/LDA,^27^ we explored the interaction of sex and obesity in exploratory models. A sensitivity analysis limiting to patients with available data for BMI was performed, with obesity defined by measured BMI ≥30 kg/m^2^. Finally, we further explored the odds of the presence of each component of VLDA in obese versus non-obese patients with adjustment on age and sex.

Statistical analyses were conducted using IBM SPSS Statistics for Windows, V.25 (IBM, Armonk, New York). All reported p values were two sided, and p values less than 0.05 were considered statistically significant.

## Results

Overall, 431 patients were analysed. The mean (SD) age was 52.4 (12.6) years, 49.3% were women.

### Obesity

Overall, 136 (31.6%) patients were obese defined as either BMI ≥30 kg/m^2^ or affirmed as obesity in FCI. Thirty-seven (8.6%) participants had missing data for either weight or height for the computation of BMI. Among patients classified as obese according to FCI, 7 out of 70 participants (10%) had BMI <30 kg/m^2^. Among the non-obese cases according to the FCI, 54 out of 324 participants (16.7%) had their measured BMI ≥30 kg/m^2^. The 136 patients defined here as obese were older, more frequently woman and had a higher number of other comorbidities compared with those who were non-obese (table 1). Compared with non-obese patients, patients with obesity had higher tender joint counts, enthesitis scores and CRP, but not swollen joint counts. In addition, PROs were significantly worse among patients with obesity than those without, including higher pain score, worse PGA, worse physical function by HAQ-DI and worse HR-QoL in all PsAID domains except skin (table 1). Rates of obesity by country are shown in online supplemental table 1.

10.1136/rmdopen-2023-003157.supp1 Supplementary data

**Table 1:** Patient characteristics of psoriatic arthritis patients with/without obesity (n=431)

	Non-obese (n=295)	Obese (n=136)
Age, years	51.5 (13.1)	54.4 (11.0) *
Female, n (%)	125 (43.3)	84 (62.2) **
Schooling, years	12.5 (4.2)	11.7 (4.6)
Duration of PsA, years	11.1 (8.3)	10.5 (7.8)
Number of other comorbidities (FCI), 0–17	1.6 (0.9)	2.4 (2.0) **
Current use of csDMARDs, n (%)	170 (61.2)	85 (66.9)
Current use of bDMARDs n (%)	173 (61.3)	76 (60.8)
Tender joints, 0–68	4.0 (8.4)	6.5 (11.7) *
Swollen joints, 0–66	2.5 (8.4)	1.7 (2.8)
Leeds enthesitis index, 0–6	0.5 (1.2)	0.9 (1.7) **
Psoriasis severity, n (%)		
No psoriasis/ limited psoriasis (<1–5%)	258 (91.8)	116 (88.6)
Extensive psoriasis (6–20%)	18 (6.4)	13 (9.9)
Very extensive psoriasis (>20)	5 (1.8)	2 (1.5)
CRP, mg/dL	1.2 (3.5)	2.3 (8.1) *
Pain, 0–10	3.8 (2.7)	4.8 (2.8) **
PGA disease activity, 0–10	2.9 (2.5)	3.5 (2.5) *
HAQ-DI, 0–3	0.5 (0.6)	0.9 (0.7) **
DAPSA	15.2 (17.0)	19.9 (18.0) *
PsAID-12, 0–10	3.0 (2.3)	4.1 (2.6) **
Pain, 0–10	3.7 (2.8)	4.7 (2.8) **
Skin, 0–10	2.5 (2.7)	3.0 (2.9)
Fatigue, 0–10	3.9 (3.0)	4.8 (3.2) **
Work or leisure activities, 0–10	3.3 (3.0)	4.8 (3.26) **
Functional capacity, 0–10	3.3 (2.9)	4.5 (3.2) **
Discomfort, 0–10	3.4 (2.9)	4.6 (3.0) **
Sleep, 0–10	2.9 (3.1)	4.1 (3.4) **
Anxiety, 0–10	2.8 (3.0)	3.5 (3.3) *
Coping, 0–10	2.5 (2.6)	3.4 (3.0) **
Embarrassment, 0–10	1.8 (2.7)	2.8 (3.3) **
Social participation, 0–10	2.1 (2.9)	3.0 (3.3) **
Depression, 0–10	1.9 (2.7)	2.9 (3.3) **

Mean (SD) shown unless specified otherwise. *p<0.05; **p<0.01 for comparisons between non-obese and obese groups.

bDMARDs, biological disease modifying anti-rheumatic drugs; CRP, C reactive protein; csDMARDs, conventional synthetic disease modifying anti-rheumatic drugs; DAPSA, Disease Activity in PSoriatic Arthritis; FCI, Functional Comorbidity Index; HAQ-DI, Health Assessment Questionnaire Disability Index; LDA, low disease activity; PGA, patient global assessment of disease activity; PsA, psoriatic arthritis; PsAID, Psoriatic Arthritis Impact of Disease.

### Remission/LDA

Congruent with our previous report, 19.5%, 14.8% and 22.7% of patients reached remission by DAPSA-REM, VLDA and patients’ opinion, respectively, while 56.4%, 51.8% and 60.2% reached LDA by DAPSA-LDA, MDA and patients’ opinion. Patient characteristics stratified by remission/LDA criteria are shown in online supplemental tables 2 and 3. Patients not having remission/LDA by indices were more frequently women, had higher number of comorbidities and had higher BMI. Demographics of patients by remission/LDA indices and patients’ opinion are shown in online supplemental tables 2 and 3.

### Association between obesity and remission/LDA

**Figure 1:**
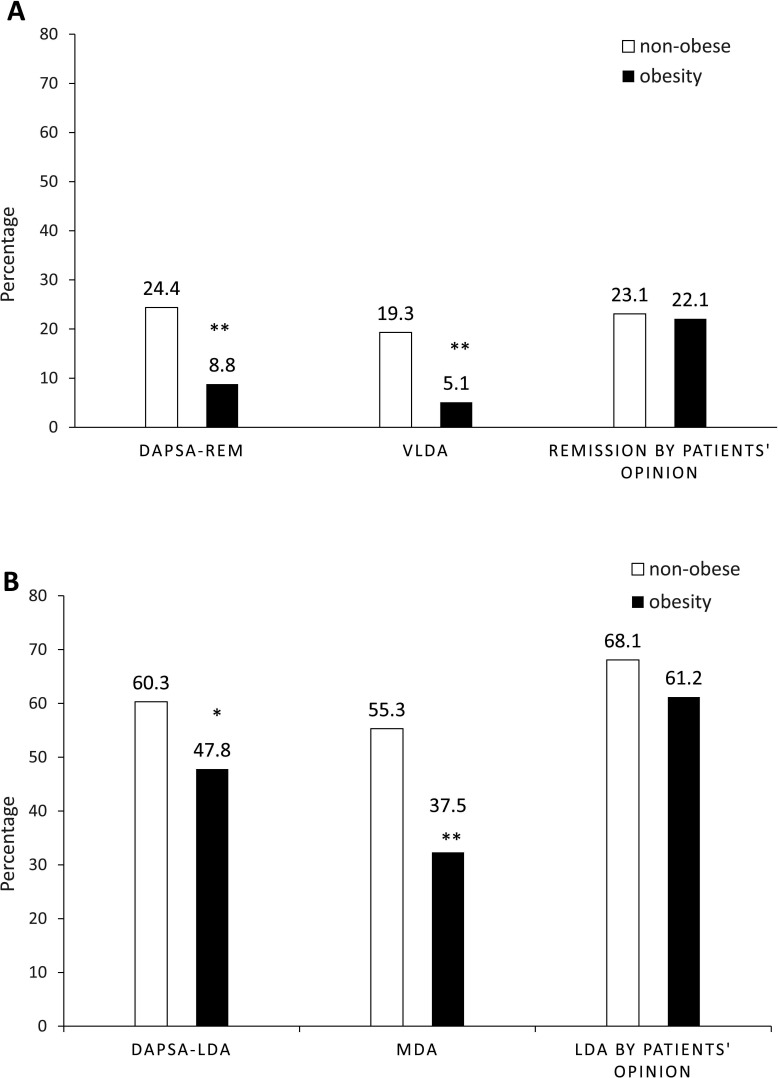
Rates of remission/ LDA for patients with obesity vs non-obese. (A) Rates of remission for patients with obesity vs non-obese. (B) Rates of low disease activity for patients with obesity vs non-obese. *Unadjusted p<0.05; **Unadjusted p<0.001; DAPSA-LDA, Low disease activity by Disease Activity in Psoriatic Arthritis; DAPSA-REM, Remission by Disease Activity in Psoriatic Arthritis; LDA, low disease activity state; MDA, minimal disease activity; VLDA, very low disease activity.

The respective unadjusted rates for remission by DAPSA-REM, VLDA and patients’ opinion in patients with obesity compared with non-obese were 8.8% vs 24.4% (p <0.001), 5.1% vs 19.3% (p<0.001) and 22.1% vs 23.1 (p=0.90). The respective rates for LDA by DAPSA-LDA, MDA and patients’ opinion were 47.8% vs 60.3% (p=0.0016), 37.5% vs 55.3% (p<0.001) and 61.2% vs 68.1% (p=0.187). Thus, there was a statistically significantly lower proportion of patients with obesity who were in remission by VLDA and DAPSA, but there was no difference in remission from the patients’ opinion (figure 1A). A similar pattern was seen for LDA states and patients’ opinions (figure 1B).

In the univariable model, obesity, female sex, current DMARD use and higher number of other comorbidities were associated with lower odds of being in remission by VLDA (online supplemental table 4). Obesity, female sex, longer duration of PsA and higher number of other comorbidities were associated with lower odds of DAPSA-REM. The associations between obesity and all models on remission/ LDA are summarised in table 2. The detail of statistical analysis of each remission/ LDA status in each model is given in online supplemental tables 4–9.

**Table 2:** Summary of odds to be in remission/LDA comparing patients with obesity versus non-obese

	Univariable model		Multivariable model*	
	OR (95% CI)	P	OR (95% CI)	P
Remission				
DAPSA-REM	**0.32 (0.16, 0.62**)	**0.001**	**0.39 (0.19, 0.80**)	**0.009**
VLDA	**0.23 (0.10, 0.55**)	**0.001**	**0.31 (0.13, 0.77**)	**0.011**
Remission by patients’ opinion	0.869 (0.504, 1.497)	0.613	–	–
LDA				
DAPSA-LDA	0.448 (0.263, 0.762)	0.003	0.728 (0.446, 1.187)	0.203
MDA	**0.50 (0.31, 0.78**)	**0.002**	**0.61 (0.38, 0.99**)	**0.045**
LDA by patients’ opinion	0.80 (0.51, 1.26)	0.333	–	–

Bold: variables statistically significantly associated with remission/ LDA of interest.

*List of adjustment variables considered were age, sex, years of schooling, duration of PsA, current use of cDMARDs, current use of bDMARDs and other comorbidities in FCI. Variables adjusted in each multivariable model were chosen from those with significant p<0.1 in univariable model as detailed in online supplemental tables 4–8.

bDMARDs, biological disease modifying anti-rheumatic drugs; csDMARDs, conventional synthetic disease modifying anti-rheumatic drugs; DAPSA, Disease Activity in PSoriatic Arthritis; FCI, Functional Comorbidity Index; LDA, low disease activity status; REM, remission; VLDA, very low disease activity; vs, versus.

In the multivariable model, obesity was statistically significantly associated with lower odds of DAPSA-REM, after adjustment for age, sex, use of b/cs-DMARDs and other comorbidities (OR 0.39; 95% CI 0.19 to 0.80; p=0.009) (table 2 and online supplemental table 4). Similarly, obesity remained statistically significantly associated with lower odds of VLDA after adjustments (table 2 and online supplemental table 1). Patients with obesity were three times less likely than those without to be in VLDA (OR 0.31; 95% CI 0.13 to 0.77; p=0.011). However, obesity was not associated with remission by patients’ opinion (table 2 and online supplemental table 6). Obesity was associated with MDA in both univariable and multivariable adjustment models (online supplemental table 7); the association of obesity and DAPSA-LDA was lost in the multivariable model (online supplemental table 8).

In the exploratory model, we did not observe a significant interaction term between sex and obesity, p for interaction were 1.00 and 0.21, respectively, for VLDA and DAPSA-REM models. Results of sensitivity analysis limiting to patients with BMI data and obesity defined as BMI ≥30 kg/m^2^ were generally consistent (online supplemental table 10).

For VLDA components, the odds of being in low pain level and good functional outcomes were lower in obese patients versus non-obese, which remained statistically significant after adjustment with age and sex (table 3).

**Table 3:** Association between obesity and probability of each component in MDA

	Univariable model		Multivariable model *	
	OR (95% CI)	P	OR (95% CI)	P
Tender joint count≤1	0.62 (0.41, 0.93)	0.021	0.71 (0.45, 1.13)	0.152
Swollen joint count≤1	0.73 (0.47, 1.13)	0.154	0.78 (0.48, 1.28)	0.328
PASI≤1 or BSA≤3%	0.68 (0.34, 1.35)	0.274	0.55 (0.25, 1.18)	0.123
HAQ≤0.5	**0.39 (0.26, 0.60**)	**<0.001**	**0.52 (0.32, 0.83**)	**0.007**
Pain≤1	**0.40 (0.23, 0.69**)	**0.001**	**0.50 (0.27, 0.91**)	**0.023**
PGA≤2	0.63 (0.42, 0.95)	0.027	0.81 (0.51, 1.28)	0.369
Tender entheseal site≤1	0.54 (0.31, 0.94)	0.030	0.64 (0.33, 1.23)	0.635

Bold: statistically significant.

*Regression model adjusted with age and sex.

bDMARDs, biological disease modifying anti-rheumatic drugs; BSA, body surface area; csDMARDs, conventional synthetic disease modifying antirheumatic drugs; DAPSA, Disease Activity in PSoriatic Arthritis; FCI, Functional comorbidity index; HAQ, Health Assessment Questionnaire; LDA, low disease activity status; PASI, Psoriasis Area and Severity Index; PGA, patient global assessment.

## Discussion

In this multicentre international sample of patients with established PsA, we found that patients with comorbid obesity had lower odds of being in remission/LDA defined by different indices, after adjustment for multiple other variables. Patients with obesity were 2.5–3 times less likely than their non-obese counterparts to be in DAPSA remission and VLDA; and 1.6 times less likely to be in MDA. However, the rates of remission by patients’ opinion and the rates of DAPSA-LDA (defined as DAPSA ≤14) were similar comparing patients with or without comorbid obesity in the multivariable-adjusted models. Compared with non-obese patients, low pain levels and good physical function were the most difficult components to be among the VDLA components, for obese patients.

Obesity is prevalent among patients with PsA and associated with higher disease activity, worse disease impact^16 17^ and possibly poorer response to treatment.^28^ Obesity represents a state of low-grade systemic inflammation, and elevated CRP has been repeatedly described across different population, across sex and age groups.^29^ Adipokines secreted by adipose tissue are increasingly recognised to regulate various immune responses. For instances, the most described adipokine, leptin, promotes pro-inflammatory signals including interleukin (IL)−1α, IL-17 and tumour necrosis factor (TNF)-α.^30^ It has been reported that PsA patients who are obese may have higher disease activity and poorer response to treatment. PsA patients who were obese had higher disease burden measured by PsAID and Routine Assessment of Patient Index Data (RAPID3).^31^ In a longitudinal cohort, PsA patients with obesity had a lower probability of achieving sustained MDA.^16^ Among patients initiating TNF inhibitor treatment, obesity was a strong predictor of not achieving Clinical Disease Activity Index remission,^17^ while weight loss was associated with a higher rate of achieving MDA.^32^ Our study findings generally expand those reported in the literature. CRP levels among our patients with obese were two times as high as those without. Once again, we observe that obesity is associated with higher pain scores, and worse physical function and HR-QoL. In addition, we showed that patients with comorbid obesity also had lower probability to be in remission. This highlights the importance of recognising and managing obesity as an important comorbidity to improve the care of patients with PsA.

Despite the lack of consensus in the instruments to define remission, achieving remission is a desirable target in the care of patients with PsA.^4^ Several index-based criteria for remission have been developed and demonstrated their ability to predict less radiographic damage among patients achieving them.^6 11 33^ This theoretically should lead to better quality of life for patients with PsA. Yet, what patients perceive as ’good status’ is less studied. We previously explored clusters of factors associated with remission and LDA in patients’ opinion and found that apart from disease impact and disease activity in different domains, chronicity/age and comorbidities were the key contributing factors.^26^ Using the same data set, we have also reported that there was only moderate agreement between patient-perceived remission and remission defined by VLDA (Kappa 0.65) or DAPSA (Kappa 0.60). Close to half of patients in self-perceived remission were not in VLDA or DAPSA remission, while a third of those in self-perceived LDA were not considered in LDA by index-based measures.^10^ In the current study, we further revealed that one of the key comorbidities, obesity was associated with high disease impact by indices on patients with PsA. However, we did not observe a statistically significant association between obesity and remission according to patients’ opinion. Obese patients reported higher disease impact but despite this they also reported that their disease was controlled. The reasons behind this are unclear. Possible reasons include overestimating swollen joints in obese patients, higher articular damage in obesity due to osteoarthritis and influences from other comorbidities. While numerous studies in the general population have reported associations between obesity and chronic pain, reduced physical activity and pain sensitisation,^34^ the mechanism underlying this observation is elusive. Mechanical, behavioural and psychological factors may play a role that requires further studies.

Exploring the individual components within VLDA, obese patients were less likely to have a low pain score and good physical function compared with non-obese. Whether obese patients may have a less stringent threshold to endorse remission requires more study. The same is true for potential psychological factors such as expectations of the target of good status that they could achieve, and how much symptoms patients may attribute to PsA or their comorbidities including obesity.^35^ Equally, the benefit of remission versus LDA in this patient population needs to be clarified. Results from this study support a recommendation to manage obesity in PsA with the hope to facilitate more successful T2T treatment and to decrease symptoms and life impact burden. The key message for physicians and healthcare providers is the awareness of the importance of shared decision-making when discussing the impact of obesity on expected treatment outcomes and the implications for disease management. For instance, higher BMI may adversely affect the volume of drug distribution, leading to insufficient dosing and reduced efficacy, particularly for TNF blockers.^28 36^ Currently, infliximab, golimumab and ustekinumab can be given as weight-based regimen, while other biologics are usually given in fixed doses.^37^ In addition to emphasis on weight reduction, adjustment of dosing of therapeutics may be considered for individual patients.

We acknowledge several limitations of our study. First, patients were recruited from tertiary referral centres and results may not be generalisable to milder PsA. The cross-sectional analysis does not allow study on changes in remission status or causality. Although we collected data for one follow-up visit, the small sample size precluded longitudinal analysis. Data from this study do not preclude obese patients having high disease activity and impact at baseline, and still have good potential to improve and be satisfied with T2T strategy overtime. We may not be able to account for variables we did not collect, such as fibromyalgia, which could be an important interaction with obesity in the models. Comorbidities in this study were collected through FCI, which was developed to predict functional outcomes rather than mortality.^19^ Finally, obesity was defined based on either BMI or physician report, which led to some discrepancies.

In conclusion, PsA patients with concurrent obesity had lower odds of being in remission by indices, highlighting the importance of managing obesity to improve the care of patients with PsA. Shared decision-making is important for an individualised target of management.

## Data Availability

Data are available upon reasonable request.
