# Supplementary material for: Association between obesity and likelihood of remission or low disease activity status in psoriatic arthritis applying index-based and patient-based definitions of remission: a cross-sectional study
Source: RMD Open. 2023 Sep 14;9(3):e003157. doi: 10.1136/rmdopen-2023-003157 (PMC10503343; doi:10.1136/rmdopen-2023-003157)
Supplement: Supplementary data [file rmdopen-2023-003157supp001.pdf]

Leung, et al. Obesity and remission, ReFlaP study

Supplementary Table 1. Rates of obesity by country

Countries	Recruited patients (N)	Rates of obesity within the country (%)
Austria	7	42.9
Brazil	29	34.5
Canada	37	43.2
Estonia	22	40.9
France	84	17.9
Germany	30	46.7
Italy	45	8.9
Romania	24	45.8
Russia	20	45.0
Singapore	28	14.3
Spain	30	20.0
Turkey	18	55.6
United Kingdom	17	35.3
United States of America	40	47.5

Leung, et al. Obesity and remission, ReFlaP study

Supplementary Table 2. Baseline characteristics of PsA patients stratified according to disease activity categories (n=431)

	High/Moderate Disease Activity (n= 188)	LDA+REM€ (n= 243)	REM€ (n= 84)	Not achieving MDA (n= 217)	MDA¥ (n= 214)	VLDA¥ (n= 64)
Age, years	52.9 (12.3)	52.0 (12.7)	53.0 (12.3)	52.4 (12.6)	52.4 (12.6)	49.6 (12.1)
Female, n (%)	113 (61.1)	96 (40.2) **	24 (28.9) **	128 (59.8)	81 (38.6) **	18 (28.6) **
Schooling, years	12.1 (4.0)	12.4 (4.6)	12.4 (5.1)	11.8 (4.2)	12.6 (4.4) *	13.0 (4.7)
Duration of PsA, years	10.1 (8.2)	11.6 (8.1)	12.9 (8.0) *	10.0 (7.8)	11.9 (8.4) *	11.6 (7.5)
Obesity, n (%)	71 (37.8)	65 (26.7) *	12 (14.3) **	85 (39.2)	51 (23.8) **	7 (10.9) **
BMI kg/m^2	28.9 (7.2)	27.9 (6.4)	26.5 (5.8) **	29.4 (6.6)	27.3 (6.8) **	26.0 (4.2) **
FCI (0-18)	2.0 (1.4)	1.7 (1.4) *	1.5 (0.9) **	2.1 (1.3)	1.7 (1.4) **	1.3 (0.7) **
Current use of csDMARDs, n (%)	109 (61.2)	146 (64.3)	44 (55.0)	130 (63.1)	125 (62.8)	32 (51.6)
Current use of bDMARDs n (%)	91 (52.3)	158 (67.8) **	56 (68.3) **	117 (57.4)	132 (65.0)	45 (73.8)
Tender joints, 0-68	10.0 (12.7)	0.7 (1.1) **	0.2 (0.5) **	9.0 (12.1)	0.4 (0.9) **	0.1 (0.3) **
Swollen joints, 0-66	4.7 (10.2)	0.4 (0.9) **	0.1 (0.3) **	4.2 (9.6)	0.3 (1.0) **	0.1 (0.3) **
Leeds enthesitis index, 0-6	1.2 (1.9)	0.1 (0.5) **	0.1 (0.4) **	1.1 (1.8)	0.1 (2.9) **	0.0 (0.2) **
CRP, mg/dL	2.8 (7.9)	0.6 (1.3) **	0.2 (0.3) **	2.1 (7.0)	1.1 (2.9)	0.8 (2.3)
Pain, 0-10	6.3 (2.1)	2.3 (1.8) **	0.8 (0.8) **	5.8 (2.3)	2.3 (2.0) **	0.5 (0.5) **
PGA, 0-10	4.8 (2.3)	1.8 (1.7) **	0.7 (0.9) **	4.9 (2.1)	1.3 (1.2) **	0.5 (0.6) **
HAQ-DI, 0-3	1.0 (0.7)	0.4 (0.5) **	0.2 (0.3) **	1.1 (0.7)	0.3 (0.4) **	0.0 (0.1) **
DAPSA	30.2 (18.7)	6.2 (3.9) **	2.0 (1.3) **	26.8 (19.2)	6.4 (5.0) **	2.3 (2.9) **
PsAID-12, 0-10	5.1 (2.2)	2.0 (1.7) **	0.8 (0.8) **	4.9 (2.1)	1.8 (1.6) **	0.7 (0.8) **

*<0.05; **<0.01; €comparison made with high/moderate disease activity; ¥comparison made with patients not achieving MDA.
Mean (SD) shown unless specified otherwise. BMI: body mass index; bDMARDs: biological disease modifying anti-rheumatic drugs; csDMARDs: conventional synthetic disease modifying anti-rheumatic drugs; CRP: C-reactive protein; DAPSA: Disease Activity in PSoriatic Arthritis; FCI: Functional comorbidity Index; HAQ-DI: Health Assessment Questionnaire Disability Index; HAD: high disease activity; LDA: low disease activity by DAPSA; PsA: psoriatic arthritis; PGA: patient global assessment of disease activity; PsAID: Psoriatic Arthritis Impact of Disease; REM: remission by DAPSA.

Leung, et al. Obesity and remission, ReFlaP study

Supplementary Table 3. Baseline characteristics of PsA patients stratified according to disease status by patients' opinion (n=431)

	Not at REM/LDA (n= 146)	Low disease activity status by patient opinion¥ (n= 257)	Remission by patient opinion¥ (n= 98)
Age, years	51.6 (13.1)	53.3 (12.3)	51.7 (12.4)
Female, n (%)	86 (60.6)	110 (43.3) **	36 (37.1) **
Schooling, years	12.2 (4.8)	12.2 (4.2)	12.2 (4.2)
Duration of PsA, years	10.5 (7.9)	11.4 (8.5)	11.3 (8.5)
Obesity, n (%)	52 (35.6)	76 (29.6)	30 (30.6)
BMI kg/m^2	29.0 (6.3)	28.4 (6.5)	29.0 (8.4)
FCI (0-18)	2.1 (1.3)	2.0 (1.7)	2.1 (2.2)
Current use of csDMARDs, n (%)	83 (60.1)	162 (67.5)	58 (63.0)
Current use of bDMARDs n (%)	75 (55.1)	154 (63.4)	61 (67.8)
Tender joints, 0-68	8.2 (11.4)	2.8 (7.1) **	3.2 (10.1) **
Swollen joints, 0-66	4.2 (9.9)	1.4 (5.1) **	0.9 (3.4) **
Leeds enthesitis index, 0-6	1.1 (1.8)	0.3 (0.9) **	0.3 (1.2)
CRP, mg/dL	1.8 (4.3)	1.5 (6.0)	1.3 (3.4)
Pain, 0-10	6.2 (2.2)	3.1 (2.4) **	2.1 (2.3) **
PGA, 0-10	6.1 (2.3)	3.3 (2.3) **	2.4 (2.5) **
HAQ-DI, 0-3	1.0 (0.7)	0.5 (0.6) **	0.3 (0.5) **
DAPSA	26.6 (17.7)	11.8 (14.4) **	9.3 (14.9) **
PsAID-12, 0-10	5.2 (2.1)	2.5 (2.0) **	1.7 (1.8) **

*p<0.05; **p<0.01; ¥comparison made with patients not achieving REM or LDA by patients' opinion.
Mean (SD) shown unless specified otherwise. BMI: body mass index; bDMARDs: biological disease modifying anti-rheumatic drugs; csDMARDs: conventional synthetic disease modifying anti-rheumatic drugs; CRP: C-reactive protein; DAPSA: Disease Activity in PSoriatic Arthritis; FCI: Functional comorbidity Index; HAQ-DI: Health Assessment Questionnaire Disability Index; HAD: high disease activity; LDA: low disease activity by DAPSA; PsA: psoriatic arthritis; PGA: patient global assessment of disease activity; PsAID: Psoriatic Arthritis Impact of Disease; REM: remission by DAPSA.

Leung, et al. Obesity and remission, ReFlaP study

Supplementary Table 4. Association between variables and probability of presence of DAPSA-remission.

	Univariable model		Multivariable model	
	OR (95% CI)	p	OR (95% CI)	p
**Obesity vs. non-obese**	**0.32 (0.16, 0.62)**	**0.001**	**0.39 (0.19, 0.80)**	**0.009**
Age	1.01 (0.99, 1.03)	0.470	-	-
**Female vs. male**	**0.36 (0.21, 0.62)**	**<0.001**	**0.40 (0.23, 0.70)**	**0.001**
Years of schooling	1.02 (0.96, 1.08)	0.626	-	-
**Duration of PsA**	**1.03 (1.00, 1.06)**	**0.033**	**1.04 (1.01, 1.07)**	**0.012**
Current csDMARDs use	0.70 (0.42, 1.16)	0.163	-	-
Current bDMARD use	1.51 (0.89, 2.56)	0.127	-	-
FCI (less obesity)	0.76 (0.58, 0.99)	0.041	0.81 (0.61, 1.08)	0.155

Bold: variables statistically significantly associated with DAPSA remission.
OR: odd ratio; CI: confidence interval; bDMARDs: biological disease modifying anti-rheumatic drugs; csDMARDs: conventional synthetic disease modifying anti-rheumatic drugs; DAPSA: Disease Activity in PSoriatic Arthritis; FCI: Functional comorbidity index; vs.: versus.

Supplementary Table 5. Association between variables and probability of presence of VLDA.

	Univariable model		Multivariable model	
	OR (95% CI)	p	OR (95% CI)	p
**Obesity vs. non-obese**	**0.23 (0.10, 0.55)**	**0.001**	**0.31 (0.13, 0.77)**	**0.011**
Age	0.98 (0.96, 1.00)	0.060	1.00 (0.97, 1.02)	0.739
**Female vs. male**	**0.35 (0.19, 0.65)**	**0.001**	**0.43 (0.22, 0.82)**	**0.011**
Years of schooling	1.02 (0.96, 1.10)	0.507	-	-
Duration of PsA	1.01 (0.98, 1.04)	0.588	-	-
Current csDMARD use	0.55 (0.31, 0.98)	0.043	0.66 (0.35, 1.24)	0.194
Current bDMARD use	2.00 (1.07, 3.71)	0.030	1.72 (0.86, 3.41)	0.125
**FCI (less obesity)**	**0.50 (0.33, 0.77)**	**0.002**	**0.56 (0.36, 0.90)**	**0.016**

Bold: variables statistically significantly associated with VLDA.
OR: odd ratio; CI: confidence interval; bDMARDs: biological disease modifying anti-rheumatic drugs; csDMARDs: conventional synthetic disease modifying anti-rheumatic drugs; VLDA: very low disease activity; FCI: Functional comorbidity index; vs.: versus.

Supplementary Table 6. Association between variables and probability of remission by patients' opinion.

	Univariable model	
	OR (95% CI)	p
Obesity vs. non-obese	0.869 (0.504, 1.497)	0.613
Age	0.993 (0.974, 1.013)	0.495
**Female vs. male**	**0.484 (0.291, 0.807)**	**0.005**
Years of schooling	1.005 (0.949, 1.064)	0.861
Duration of PsA	1.018 (0.990, 1.047)	0.220
Current csDMARD use	0.975 (0.587, 1.621)	0.922
Current bDMARD use	1.390 (0.833, 2.317)	0.207
FCI (less obesity)	1.045 (0.887, 1.232)	0.596

Bold: variables statistically significantly associated with remission by patients' opinion.
OR: odd ratio; CI: confidence interval; bDMARDs: biological disease modifying anti-rheumatic drugs; csDMARDs: conventional synthetic disease modifying anti-rheumatic drugs; DAPSA: Disease Activity in PSoriatic Arthritis; FCI: Functional comorbidity index.

Leung, et al. Obesity and remission, ReFlaP study

Supplementary Table 7. Association of variables with probability of Minimal Disease Activity (MDA)

	Univariable model		Multivariable model	
	OR (95% CI)	p	OR (95% CI)	p
**Obesity vs. non-obese**	**0.50 (0.31, 0.78)**	**0.002**	**0.61 (0.38, 0.99)**	**0.045**
Age	1.00 (0.98, 1.02)	1.00	-	-
**Female vs. male**	**0.41 (0.27, 0.62)**	**<0.001**	**0.44 (0.28, 0.67)**	**<0.001**
Years of schooling	1.04 (0.99, 1.09)	0.113	-	-
**Duration of PsA**	**1.03 (1.00, 1.06)**	**0.022**	**1.04 (1.01, 1.07)**	**0.007**
Current csDMARD use	1.03 (0.67, 1.57)	0.903	-	-
Current bDMARD use	1.32 (0.87, 1.00)	0.194	-	-
FCI (less obesity)	0.81 (0.68, 0.97)	0.019	0.85 (0.70, 1.03)	0.101

Bold: variables statistically significantly associated with MDA.
OR: odd ratio; CI: confidence interval; Bold: statistically significant; bDMARDs: biological disease modifying anti-rheumatic drugs; csDMARDs: conventional synthetic disease modifying anti-rheumatic drugs; FCI: Functional comorbidity index; MDA: minimal disease activity; vs.: versus.

Supplementary Table 8. Association of variables with probability of presence of LDA by DAPSA

	Univariable model		Multivariable model	
	OR (95% CI)	p	OR (95% CI)	p
Obesity vs. non-obese	0.448 (0.263, 0.762)	0.003	0.728 (0.446, 1.187)	0.203
Age	0.994 (0.977, 1.010)	0.466	-	-
**Female vs. male**	**0.437 (0.286, 0.668)**	**<0.001**	**0.449 (0.287, 0.702)**	**<0.001**
Years of schooling	1.017 (0.970, 1.067)	0.486	-	-
Duration of PsA	1.023 (0.997, 1.050)	0.084	1.027 (0.999, 1.056)	0.056
Current csDMARDs use	1.219 (0.795, 1.870)	0.364	1.592 (0.994, 2.548)	0.053
**Current bDMARD use**	**1.991 (1.302, 3.044)**	**0.001**	**2.178 (1.381, 3.437)**	**0.001**
FCI less obesity	0.829 (0.700, 0.981)	0.029	0.880 (0.735, 1.053)	0.162

Bold: variables statistically significantly associated with at least LDA by DAPSA (DAPSA ≤14).
OR: odd ratio; CI: confidence interval; bDMARDs: Bold: statistically significant; biological disease modifying anti-rheumatic drugs; csDMARDs: conventional synthetic disease modifying anti-rheumatic drugs; DAPSA: Disease Activity in PSoriatic Arthritis; FCI: Functional comorbidity index; LDA: low disease activity status; vs.: versus.

Leung, et al. Obesity and remission, ReFlaP study

Supplementary Table 9. Association between variables and probability of LDA by patients' opinion.

	Univariable model	
	OR (95% CI)	p
Obesity vs. non-obese	0.80 (0.51, 1.26)	0.333
Age	1.01 (1.00, 1.03)	0.107
**Female vs. male**	**0.62 (0.41, 0.95)**	**0.027**
Years of schooling	1.02 (1.00, 1.07)	0.491
Duration of PsA	1.02 (1.00, 1.05)	0.150
Current csDMARD use	1.46 (0.95, 2.25)	0.089
Current bDMARD use	1.24 (0.81, 1.90)	0.321
FCI (less obesity)	0.93 (0.86, 1.15)	0.931

Bold: variables statistically significantly associated with remission by patients' opinion.
OR: odd ratio; CI: confidence interval; bDMARDs: biological disease modifying anti-rheumatic drugs; csDMARDs: conventional synthetic disease modifying anti-rheumatic drugs; DAPSA: Disease Activity in PSoriatic Arthritis; FCI: Functional comorbidity index.

Supplementary Table 10. Summary of odds to be in remission/LDA comparing patients with BMI≥30 vs. BMI<30 (n=394). (n=394 with complete BMI data)

	Univariable model		Multivariable model[¥]	
	OR (95% CI)	p	OR (95% CI)	p
Remission				
**DAPSA-REM**	**0.33 (0.17, 0.64)**	**0.001**	**0.39 (0.19, 0.81)**	**0.011**
**VLDA**	**0.27 (0.12, 0.61)**	**0.002**	**0.35 (0.14, 0.87)**	**0.024**
Remission by patients' opinion	0.94 (0.56, 1.59)	0.824	-	-
LDA				
DAPSA-LDA	0.72 (0.46, 1.10)	0.130	-	-
**MDA**	**0.47 (0.30, 0.73)**	**0.001**	**0.55 (0.34, 0.89)**	**0.015**
LDA by patients' opinion	0.72 (0.46, 1.10)	0.130	-	-

Bold: variables statistically significantly associated with remission/ LDA of interest.
[¥] List of adjustment variables considered were age, sex, years of schooling, duration of PsA, current use of csDMARDs, current use of bDMARDs and other comorbidities in FCI. Variables adjusted in each multivariable model were chosen from those with significant p<0.1 in univariable model as detailed in Supplementary Tables 4-8.
OR: odd ratio; CI: confidence interval; bDMARDs: Bold: statistically significant; biological disease modifying anti-rheumatic drugs; csDMARDs: conventional synthetic disease modifying anti-rheumatic drugs; DAPSA: Disease Activity in PSoriatic Arthritis; FCI: Functional comorbidity index; LDA: low disease activity status; REM: remission; VLDA: very low disease activity; vs. versus.
